# Identification of an anaerobic bacterial consortium that degrades roxarsone

**DOI:** 10.1002/mbo3.1003

**Published:** 2020-02-13

**Authors:** Yasong Li, Yaci Liu, Zhaoji Zhang, Yuhong Fei, Xia Tian, Shengwei Cao

**Affiliations:** ^1^ Institute of Hydrogeology and Environmental Geology Chinese Academy of Geological Sciences Shijiazhuang China; ^2^ Key Laboratory of Groundwater Remediation of Hebei Province and China Geological Survey Shijiazhuang China

**Keywords:** anaerobic bacterial community, concentration gradients, growth curve, *Proteiniclasticum*, roxarsone

## Abstract

The degradation of roxarsone, an extensively used organoarsenic feed additive, occurs quickly under anaerobic conditions with microorganisms playing an important role in its degradation. Here, an anaerobic bacterial consortium that effectively degraded roxarsone was isolated, and its degradation efficiency and community changes along a roxarsone concentration gradient under anaerobic conditions were assessed. We used batch experiments to determine the roxarsone degradation rates, as well as the bacterial community structure and diversity, at initial roxarsone concentrations of 50, 100, 200, and 400 mg/kg. The results showed that roxarsone was degraded completely within 28, 28, 36, and 44 hr at concentrations of 50, 100, 200, and 400 mg/kg, respectively. The anaerobic bacterial consortium displayed considerable potential to degrade roxarsone, as the degradation rate increased with increasing roxarsone concentrations. Roxarsone promoted microbial growth, and in turn, the microorganisms degraded the organoarsenic compound, with the functional bacterial community varying between different roxarsone concentrations. *Lysinibacillus*, *Alkaliphilus*, and *Proteiniclasticum* were the main genera composing the roxarsone‐degrading bacterial community.

## Graphical abstract

Roxarsone (ROX) is an organoarsenic animal drug used in production as a feed additive to increase weight gain and improve feed efficiency. This study revealed the close relationship between ROX and microbial growth. A stable anaerobic bacterial consortium that effectively degraded ROX was identified. The bacterial community, growth curve, and degradation ability at different ROX concentrations were assessed.

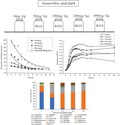

## 1 INTRODUCTION

Because of the low toxicity of roxarsone (3‐nitro‐4‐hydroxybenzylarsonic acid, ROX) and its ability to promote livestock growth, as well as its antibacterial properties, it is widely used in many countries as a feed additive (Anderson, 1983; Green & Clausen, 2005; Shelver, 2011; Yang et al., 2016) for nearly 60 years (Kowalski & Reid, 1975). Since it is found that the arsenic concentration of rivers and riverbed silt flowing through areas where livestock are concentrated is higher than the average concentration in the United States, the arsenic pollution caused by ROX as a feed additive has received increasing attention worldwide. Approximately 80% of the additive is discharged to the environment in poultry urine and excrement and degraded into more toxic compounds such as As(V) and As(III). Various arsenic speciation forms can be detected in animal manure or ROX amended soil, such as As(V), As(III), HAPA (3‐amino‐4‐hydroxy benzyl arsonic acid), MMA (methyl arsonic acid), DMA (dimethyl arsonic acid), and other arsenic species (Cortinas et al., 2006; Garbarino, Bednar, Rutherford, Beyer, & Wershaw, 2003; Ji, Shi, Kong, & Lu, 2016; Liu, Zhang, Li, Wen, & Fei, 2017a; Yang et al., 2016; Yao et al., 2013).

When the poultry litter is used as an organic fertilizer, arsenic pollution is likely to occur (Walrod, Burriss, Blue, Beck, & Atwood, 2016); for example, in 212 samples of animal manure‐based compost collected in China, 13.7% exceeded arsenic limits (Yang et al., 2017). ROX is easily biodegraded to HAPA, but HAPA persists for long periods of time in the environment, increasing the risk of arsenic contamination (Shi, Wang, Yuan, & Hu, 2014; Zhang, Wang, Yuan, & Hu, 2014). ROX can be transferred from the diet of chickens to rice plants, and soil attributes govern the phytoavailability of ROX metabolites to rice plants (Yao et al., 2019, 2016). Seasonal stratified analyses by poultry type strongly suggest that the historical use of arsenic‐based poultry drugs contributes to arsenic exposure in the population of the United States (Nigra, Nachman, Love, Grau‐Perez, & Navas‐Acien, 2017). Similarly, in Chinese province of Guangdong, both ROX and inorganic arsenic are detected at elevated levels in the chicken tissues from live poultry markets, particularly liver, and the overall health risk from dietary exposure to inorganic arsenic associated with chicken consumption is rather high (Hu, Zhang, Cheng, & Tao, 2017). Therefore, litter applied to soil as organic fertilizer can release ROX and arsenic into soil and groundwater, affecting both the environment and human health (Chen, 2007; Fisher, Yonkos, & Staver, 2015; Jackson et al., 2003; Morrison, 1969; Oyewumi & Schreiber, 2017; Wang, Chen, Sun, Gao, & Yu, 2006a; Wang & Liao, 2005).

Because of the potential food safety and environmental risks, in 1999, 2011, and 2013, the European Union, Canada, and the United States, respectively, announced that they were scrapping or discontinuing ROX use. However, it continues to be used in many other countries (Fu, He, Gong, Blaney, & Zhou, 2016). In 2003, China used 1,200 tons of ROX between the livestock and poultry industry (Zhu, 2013), and together, these animals produced 3.19 billion tons of manure which could contribute to the release of arsenic species into the environment (Wang, Ma, et al., 2006b). The China pharmacopoeia bureau issued a strong suggestion in June 2017 discouraging the use of ROX, but most livestock and poultry breeding companies continue to use the chemical.

Degradation can occur under both aerobic and anaerobic conditions (Cortinas et al., 2006; Guzmán‐Fierro et al., 2015; Mafla et al., 2015; Stolz et al., 2007), and the rate of degradation is related to the initial ROX concentration in soil (Liu, Zhang, Li, Wen, et al., 2017a). Degradation is faster in anaerobic conditions than in aerobic conditions, with ROX being completely degraded after 48 hr of dark anaerobic incubation, while only 79.9% and 94.5% were degraded after 288 hr of dark aerobic and light aerobic incubation, respectively (Liu, Zhang, Li, Wen, et al., 2017a). Light and microbial action are the main factors responsible for ROX degradation, which is also controlled by environmental factors such as moisture, temperature, and the organic content of the vadose zone (Fu, Blaney, & Zhou, 2017; Katherine et al., 2017; Sun, 2012). Some ROX‐related bacteria have been reported (Stolz et al., 2007); however, the ROX‐degrading bacterial community of anaerobic conditions and its degradation ability at different ROX concentrations have not been completely described.

This paper isolated a stable anaerobic bacterial consortium that effectively degraded ROX and evaluated its response to the initial ROX concentration through high‐throughput sequencing. Meanwhile, the ROX degradation rates of the stable bacterial consortium at different ROX concentrations under anaerobic conditions were determined. Ultimately, the relationship between ROX and the bacteria was revealed.

## 2 MATERIALS AND METHODS

### 2.1 Poultry litter samples

The poultry litter used for experiments was taken from a chicken farm (36°07′N, 115°49′E) in Yanggu County, northern Shandong plain, China. The ROX concentration of the poultry diet (34 mg/kg) was within the recommended concentration range (50 mg/kg). There were approximately 15,000 broiler chickens on the farm, and the poultry litter was perennially stored in the open air and regularly collected to use as fertilizer. The collected litter for experiments was stored at 4°C until further analysis. The collected litter was determined to have a concentration of 11.3 mg/kg of HAPA and no ROX, likely because of the rapid degradation rate of ROX.

### 2.2 Enrichment of microbial communities degrading ROX

The basal medium consisted of (per L) 4.2 g of NaHCO_3_, 0.095 g of MgCl_2_, 0.5 g of yeast extract, 10 mM lactate (Stolz et al., 2007), 10 ml of trace elements, and a vitamin mix. The pH was adjusted to 7.3. To prepare the slurry used for experiments, 5 g of chicken litter was suspended in 100 ml of sterile basal medium.

The mixed solution was dispensed into a 50 ml headspace bottle containing 40 ml of medium, 1 ml of litter slurry, and 200 mg/kg ROX, aerated with oxygen‐free N_2_, sealed, and incubated at 37°C in the dark. All experiments were performed in triplicate. The solution changed from yellow to colorless after 24 hr, and 1 ml of the solution was then transferred into a new 50‐ml headspace bottle containing 40 ml of medium and 200 mg/kg ROX. This solution was then incubated for 24 hr under the same culture conditions. The above steps were repeated 10 times to attain a stable microbial community that effectively degraded ROX, which was then used as the inoculum in the next experiment.

### 2.3 ROX degradation along a concentration gradient under anaerobic conditions

To evaluate the ability of the microbial community to degrade ROX along a concentration gradient, a 150‐ml headspace bottle was filled with 150 ml of basal mineral medium and 1 ml of a mixed bacteria solution, which was obtained from the above experiment. Four ROX concentrations (50, 100, 200, and 400 mg/kg) were examined in batch experiments. In addition, a blank control was prepared without the addition of ROX, which was compared with the samples containing ROX to determine its influence on the diversity and abundance of the microbial community. Each test was conducted in triplicate. The medium and headspace were aerated with oxygen‐free N_2_, sealed, and incubated at 37°C in the dark. Aliquots (1 ml) were taken every 4 hr and filtered through a 0.22‐µm millipore membrane for ROX analysis. Aliquots (1 ml) were taken every 2 hr to assess cell growth by measuring the optical density at 600 nm.

At the end of the anaerobic incubation period, the microorganisms from different ROX concentrations were harvested by filtering the solution through a 0.22‐µm millipore membrane. Then, the DNA was extracted for high‐throughput sequencing.

### 2.4 ROX analysis

ROX (purity > 99%) was purchased from Dr.Ehrenstorfer. The ROX concentration was measured by high‐performance liquid chromatography (LC‐20AD; Shimadzu Corporation) with a diode array detector (SPD‐M20A). The Shimpak‐ODS C18 column (250 mm × 4.6 mm, 5 μm) was used. The mobile phase consisted of 0.02 M KH_2_PO_4_, 10% (v/v) formic acid, and methanol (20:20:60, by volume), at a flow rate of 1.0 ml/min. The column was maintained at 30°C. The detection wavelength was 267 nm, and the sample size was 20 μl.

### 2.5 Kinetic model

First‐order kinetic models are often used to describe the degradation of organic compounds (Gao, Zhang, Chen, Zheng, & Liu, 2010). Therefore, the biotransformation of ROX was characterized by a first‐order kinetic model.

### 2.6 Amplification and sequencing of bacterial 16S rRNA genes

High‐throughput sequencing was conducted at Shanghai Personal Biotechnology Co., Ltd. Total bacterial genomic DNA samples were extracted using Fast DNA SPIN extraction kits (MP Biomedicals), following the manufacturer's instructions, and stored at −20°C prior to further analysis. The quantity and quality of extracted DNA were measured using a NanoDrop ND‐1000 spectrophotometer (Thermo Fisher Scientific) and agarose gel electrophoresis, respectively. PCR amplification of the bacterial 16S rRNA genes V4–V5 region was performed using the forward primer 515F (5′–GTGCCAGCMGCCGCGGTAA–3′) and the reverse primer 907R (5′–CCGTCAATTCMTTTRAGTTT–3′). Sample‐specific 7‐bp barcodes were incorporated into the primers for multiplex sequencing. PCR amplicons were purified with Agencourt AMPure Beads (Beckman Coulter) and quantified using the PicoGreen dsDNA Assay Kit (Invitrogen). After the individual quantification step, amplicons were pooled in equal amounts, and pair‐end 2 × 300 bp sequencing was performed using the Illlumina MiSeq platform with the MiSeq Reagent Kit v3.

### 2.7 High‐throughput sequence analysis

The Quantitative Insights Into Microbial Ecology (QIIME, v1.8.0) pipeline was employed to process the sequencing data, as previously described (Caporaso et al., 2010). Briefly, raw sequencing reads with exact matches to the barcodes were assigned to respective samples and identified as valid sequences. Low‐quality sequences were excluded. Paired‐end reads were assembled using FLASH (Magoc & Salzberg, 2011). After chimera detection, the remaining high‐quality sequences were clustered into operational taxonomic units (OTUs) at 97% sequence identity by UCLUST (Edgar, 2010). The most abundant sequence of each cluster was picked to be the representative sequence. OTU taxonomic classification was conducted by BLAST searching the representative sequences set against the Greengenes Database (DeSantis et al., 2006). An OTU table was further generated to record the abundance of each OTU in each sample and the taxonomy of these OTUs.

Sequence data analyses were mainly performed using QIIME and R packages (v3.2.0). OTU‐level alpha diversity indices, such as the Chao1 richness estimator (Chao, 1984), ACE metric (abundance‐based coverage estimator; Chao & Yang, 1993), Shannon diversity index (Shannon, 1948), and Simpson index (Simpson, 1949), were calculated using the OTU table in QIIME. Principal component analysis (PCA) was also conducted based on genus‐level compositional profiles (Ramette, 2007). The taxonomic compositions and abundances were visualized using MEGAN (Huson, Mitra, Ruscheweyh, Weber, & Schuster, 2011) and GraPhlAn (Altschul et al., 1997). A Venn diagram was generated to visualize the shared and unique OTUs among samples using R package “VennDiagram,” based on the occurrence of OTUs across samples/groups regardless of their relative abundance (Zaura, Keijser, Huse, & Crielaard, 2009). Partial least squares‐path modeling (PLS‐PM) was used to explore the relationships between bacterial communities and ROX concentration by using the R package (Sanchez, 2013).

## 3 RESULTS

### 3.1 ROX degradation at different concentrations

**Figure 1:**
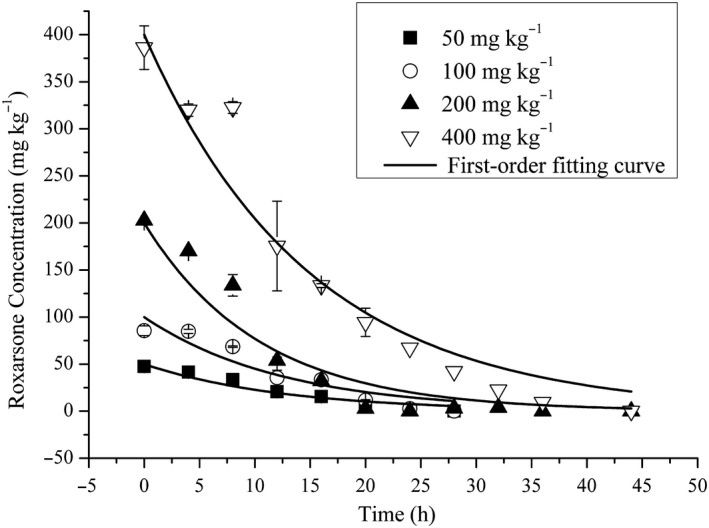
ROX degradation and the first‐order fitting curves of different ROX concentrations under anaerobic conditions

ROX was degraded rapidly under anaerobic conditions and was not detected in the blank control. ROX degradation over time and the first‐order fitting curves of different concentrations are shown in Figure 1. The kinetics of ROX degradation using the first‐order model are shown in Table 1. At concentrations of 50, 100, 200, and 400 mg/kg, ROX was degraded completely within 28, 28, 36, and 44 hr, respectively, and the half‐lives were 8.9, 8.8, 7.3, and 11.55 hr, respectively. The marginally longer half‐life at 400 mg/L when compared to the other concentrations indicates that, under anaerobic conditions, ROX was degraded more rapidly at higher initial concentrations. There were no significant differences between the degradation rates of different concentrations of ROX over time (*p* > .05; Figure 1), when compared with a one‐way analysis of variance (ANOVA) using SPSS Statistics for Windows (Fu et al., 2017). This indicates that increasing the ROX concentration had minimal effect on ROX degradation and further illustrates that anaerobic microbes possess great potential to degrade ROX.

**Table 1:** First‐order kinetic parameters of ROX degradation

ROX concentration C_0_ (mg/kg)	Rate constant k (/hr)	Regression coefficient *r* ^2^	Half‐life t_1/2_ (hr)
50	0.078	0.93	8.9
100	0.079	0.89	8.8
200	0.095	0.93	7.3
400	0.060	0.95	11.55

**Figure 2:**
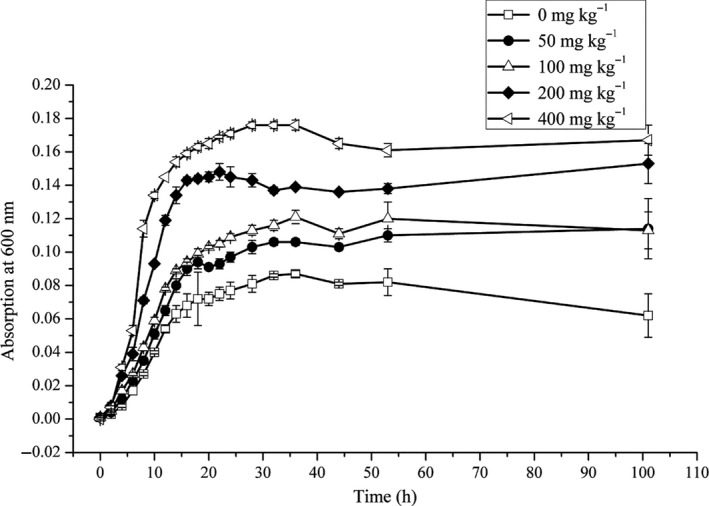
Microbial growth curves in liquid medium containing different ROX concentrations under anaerobic conditions

The microorganisms in all media grew rapidly, and a lag period was hardly observed. Cell numbers increased exponentially, reaching the stationary phase by 20 hr (Figure 2). The ROX‐free medium had the lowest microbial biomass, with microorganisms growing faster as ROX concentrations increased. The cell density at 400 mg/kg ROX was nearly threefold higher than that at 0 mg/kg. These results revealed a relationship between ROX and the microorganisms. The presence of ROX did not inhibit microbial growth; instead, it promoted microbial growth, and in turn, the microorganisms degraded ROX.

### 3.2 Alpha diversity of the bacterial community

**Figure 3:**
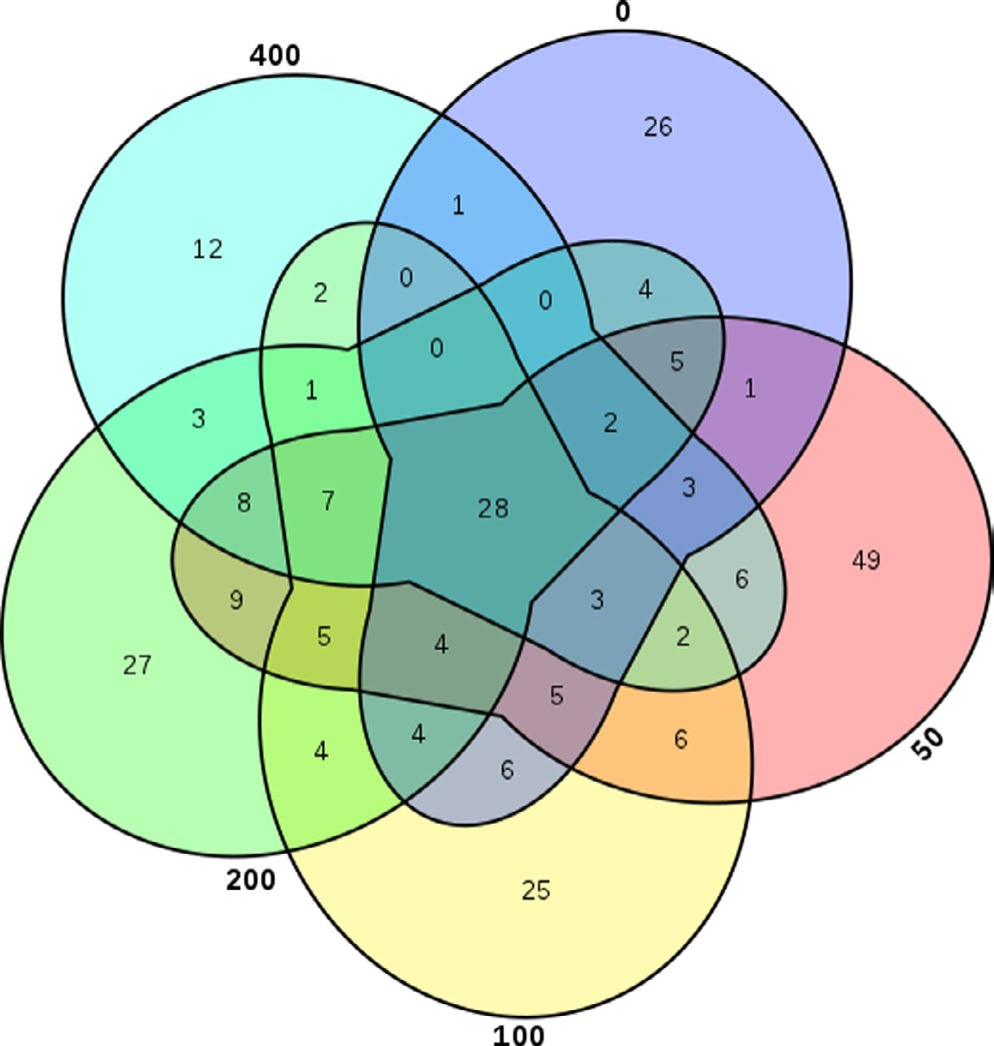
Venn diagram showing shared OTUs among samples of different ROX concentrations

**Figure 4:**
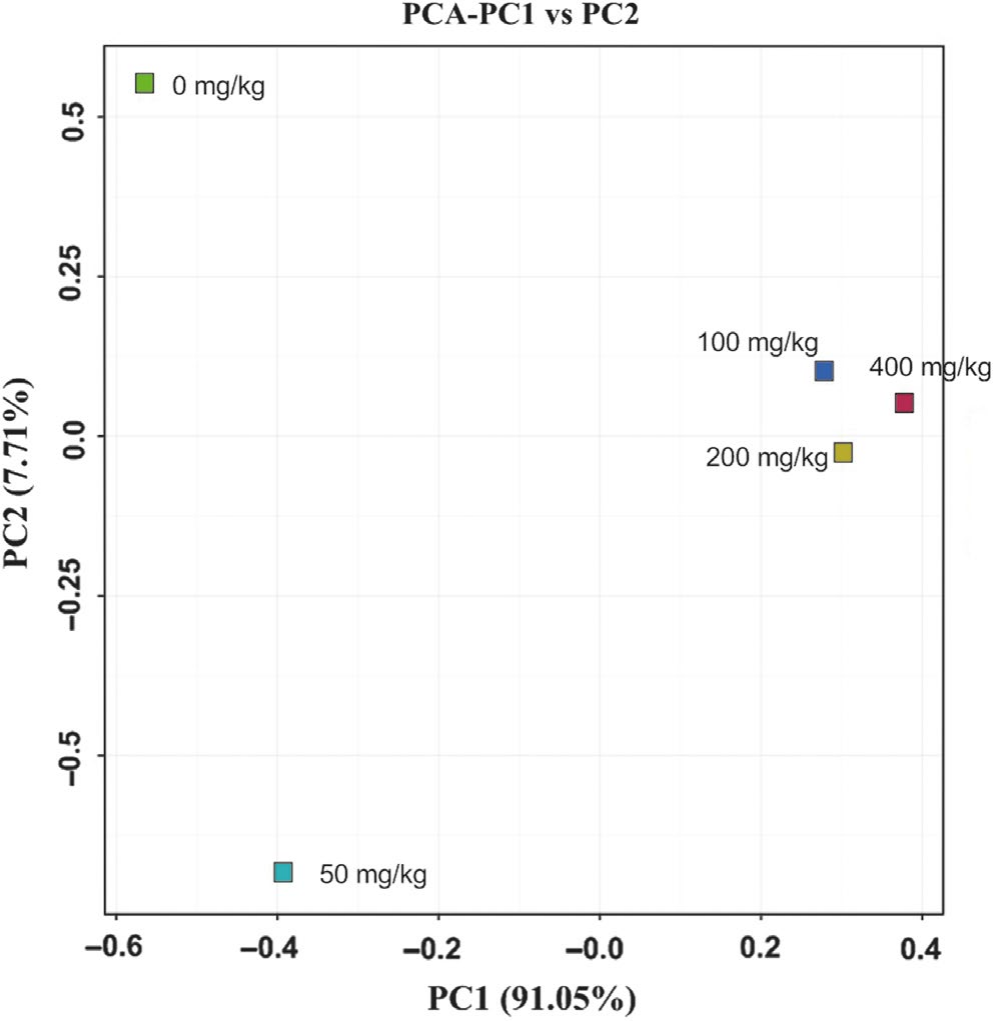
Principal component analysis (PCA) biplot of five samples under different ROX concentrations. Each coordinate axis is the interpretation proportion of the sample differences in the original data

Analysis by high‐throughput sequencing resulted in 60,282 high‐quality, nonplastid, and partial sequences were obtained for the five samples after quality control. The alpha diversity indices are shown in Table 2. A variety of taxa were observed at the 97% OTU level, with 43–71 predicted OTUs (based on Chao1). The greater the Simpson and Shannon indices are, the higher the diversity of the community is. According to the variation trend of Simpson and Shannon indices, with increasing ROX concentrations, the bacterial diversity increased initially (from 0 mg/kg to 50 mg/kg and 100 mg/kg) and then decreased (200 mg/kg, 400 mg/kg). The bacterial diversity of the sample with 400 mg/kg ROX was the lowest among the five samples. To a point, increasing ROX concentrations increased bacterial diversity; however, once the concentration reached 200 mg/kg some bacterial populations were inhibited resulting in decreased bacterial diversity and the enrichment of ROX tolerant bacteria. Creation of a Venn diagram (Figure 3) revealed the unique and shared OTUs for each of the five samples. Overall, 28 OTUs were common to all five samples. A principal component analysis (PCA) biplot of the five samples on genus level (Figure 4) showed that the samples with 100, 200, and 400 mg/kg ROX were clustered together, whereas this distance was relatively far from the 0 mg/kg and 50 mg/kg samples. The interpretation proportion of *x* axis is 91.05%, illustrating that bacterial communities developed under high ROX concentrations were different from those with no or with low ROX (50 mg/kg). In addition, the partial least squares‐path model (PLS‐PM) was constructed to integrate the complex interrelationships among ROX and bacterial communities. The PLS‐PM is represented here with a goodness‐of‐fit (GoF) value of 0.546. According to the PLS‐PM, ROX concentration exerted a significant positive direct effect on the micro community (*F* = 0.907, *R* ^2^ < 0.01), further indicating that ROX can affect the composition of bacterial communities.

**Table 2:** Alpha diversity indices at a 97% similarity level of 16S rRNA gene fragments

ROX concentration (mg/kg)	Reads	OTU	Chao1	ACE	Simpson	Shannon
0	12,527	93	46	50.28	0.54	1.7510
50	11,760	145	71	83.72	0.64	2.2970
100	11,104	114	53	53.00	0.68	2.1050
200	12,690	104	56	66.78	0.59	1.9409
400	12,201	79	43	52.96	0.49	1.4769

### 3.3 Bacterial community composition and relative abundances

**Figure 5:**
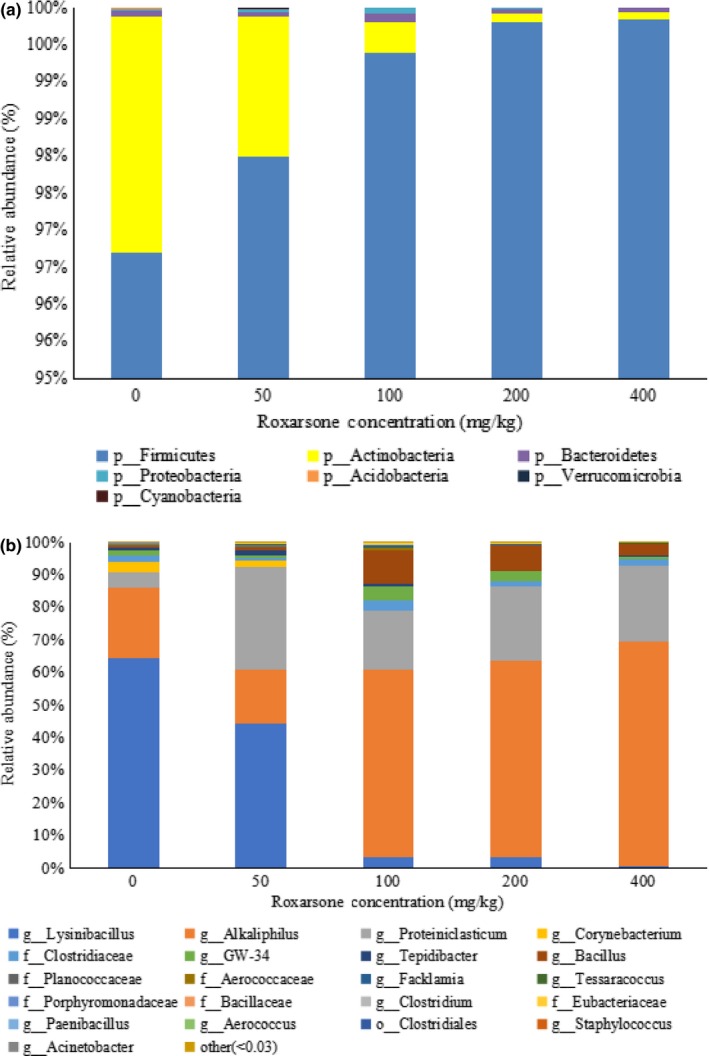
Relative abundances of bacterial taxa recovered from each sample at different ROX concentrations. (a) The relative abundance of the phylum and (b) the relative abundance of the genus

There were seven phyla identified in the five samples, with *Firmicutes* being the major phylum in each sample, with relative abundances of 96.70%–99.85% (Figure 5a). The bacterial community composition and relative abundance of each sample at the genus level (Figure 5b) identified the functional bacterial communities that degraded ROX differed at different ROX concentrations. Within the *Firmicute* phylum, *Lysinibacillus*, *Alkaliphilus,* and *Proteiniclasticum* were identified as the essential ROX‐degrading microbes. *Lysinibacillus* was the dominant genus in both the samples without ROX (64%) and with 50 mg/kg ROX (44%). However, the relative abundance of *Lysinibacillus* decreased sharply in the samples with 100, 200, and 400 mg/kg ROX (0.8%–3%), suggesting that *Lysinibacillus* could grow at lower ROX concentrations, while its growth was inhibited severely at higher concentrations. *Alkaliphilus* and *Proteiniclasticum* were the dominant genera at higher ROX concentrations (>50 mg/kg). The bacterial diversity and abundance of samples with 100, 200, and 400 mg/kg ROX were similar. In these three samples, the relative abundances of the predominant genus, *Alkaliphilus*, were 58%–68%.

## 4 DISCUSSION

The results of ROX biodegradation at different concentrations illustrated that anaerobic microbes possess great potential for this process. Similar results were obtained in a study of ROX degradation at different ROX concentrations (0, 50, 100, and 200 mg/kg) in soil, and it was found that the degradation rate increased with increasing concentrations, further highlighting the considerable potential of soil microbes to degrade ROX (Liu, Zhang, Li, & Fei, 2017b). The presence of ROX promoted microbial growth, and in turn, the microorganisms degraded ROX. These results are consistent with previous observations in which the addition of ROX did not inhibit, but rather stimulated, the growth of the anaerobe *Shewanellaoneidensis* MR‐1 (Chen, Ke, Liang, Liu, & Wang, 2016). A previous study also showed that an isolated aerobic bacterial consortium degraded ROX and that the growth rate of the aerobic bacterial consortium was 1.4‐fold higher in the presence of ROX (Guzmán‐Fierro et al., 2015).

ROX has a hazardous effect on the native microbial community diversity and metabolic activity of soil and significantly affects overall microbial activity in soil (Jiang, Li, Wang, Li, & Wang, 2013; Mangalgiri, Adak, & Blaney, 2015). An analysis of fluorescein diacetate hydrolysis in soil found that ROX does not exert acute toxicity on soil microbes; however, fluorescein diacetate hydrolysis was inhibited gradually with the release of inorganic arsenic (Liang, Ke, Chen, Liu, & Chen, 2014). Measuring the half‐maximal inhibitory and half‐maximal effective concentrations of the As(V)‐ and As(III)‐bearing photodegradates of ROX exhibited 10‐fold higher toxicity than ROX itself, which was attributed to the improved membrane permeability of the inorganic arsenicals (Zhang, Xu, Han, Sun, & Yang, 2015). The toxicity for eukaryotic or prokaryotic cells is primarily caused by ROX biodegradation by microorganisms (Mafla et al., 2015). Therefore, in the present study, the inhibition of some soil bacteria might be from the arsenic compounds released following ROX degradation. The increasing ROX concentrations enriched the bacteria that metabolized ROX and promoted the growth of these same bacteria.

*Firmicutes* was the major phylum detected in all of the samples in this study. There have been some previous reports on the relationship between *Firmicutes* and ROX. *Firmicutes* represents the most abundant phylum in soils treated with ROX, with its abundance increasing as ROX concentrations increase, implying that *Firmicutes* abundance might be related either directly to ROX or to arsenic compounds released during its degradation (Liu, Zhang, Li, & Fei, 2017b). Similarly, *Firmicutes* were the predominant group in a microbial consortium that degraded ROX (Guzmán‐Fierro et al., 2015).

*Lysinibacillus* was inhibited severely by higher ROX concentrations. A similar phenomenon occurs in *Bacillus* at lower arsenic concentrations. *Bacillus cereus* EA5 cells effectively removed arsenic from aqueous solutions containing up to 15 mg/L arsenic, while higher concentrations led to arsenic accumulation and a dramatic decrease in the efficiency of its adsorption (Mohamed & Farag, 2015). Arsenic stress causes negative cell responses, such as a decrease in surface area and shrinking. The *Lysinibacillus* strain B1‐CDA showed potential to bioremediate arsenic compounds from contaminated water, forming a long chainlike structure following arsenic exposure (Rahman et al., 2014). The carboxyl groups of the glutamic acid residues in peptidoglycan are the major sites of metal deposition (Zouboulis, Lazaridis, Karapantsios, & Matis, 2010). *Alkaliphilus oremlandii* sp. nov. strain OhILAs is capable of transforming ROX (Fisher et al., 2008). In previous studies, *Alkaliphilus* was shown to be closely correlated with the presence of arsenic and ROX (Liu, Zhang, Li, & Fei, 2017b), which aligns with our own results showing *Alkaliphilus* to be the predominant genus of the bacterial community that degraded ROX. However, to our knowledge, we are the first to report the relationship between *Proteiniclasticum* and arsenic, although further studies are needed to reveal the function of *Proteiniclasticum* in arsenic transformation.

## 5 CONCLUSIONS

This study revealed the close relationship between ROX and microbial growth and will facilitate future studies of ROX biodegradation. There are two main causes of different degradation rates in relationship to different concentrations of ROX. First, changes in the composition of the bacterial consortium occurred with different ROX concentrations (50 mg/kg ~ 400 mg/kg). *Lysinibacillus* was the dominant genus in samples with either no ROX (64%) or 50 mg/kg ROX (44%). *Alkaliphilus* and *Proteiniclasticum* were the dominant genera as ROX concentrations increased (>50 mg/kg). Second, the presence of ROX promoted the growth of functional microorganisms, and microorganisms grow faster as ROX concentrations increased from 0 mg/kg to 400 mg/kg, resulting in a nearly 3‐fold increase in cell density from samples with no ROX to 400 mg/kg ROX. Moreover, a stable anaerobic bacterial consortium that effectively degraded ROX was identified, and it is the first to report the relationship between *Proteiniclasticum* and arsenic.

## CONFLICT OF INTERESTS

None declared.

## AUTHOR CONTRIBUTION

Yasong Li: Writing‐original draft. Yaci Liu: Writing‐review & editing. Zhaoji Zhang: Supervision. Yuhong Fei: Data curation. Xia Tian: Formal analysis. Shengwei Cao: Formal analysis.

## ETHICS STATEMENT

None required.

## Data Availability

All data generated or analyzed during this study are included in this published article.
